# Thrombocytopenia secondary to iron deficiency anemia responding to iron therapy

**DOI:** 10.1002/ccr3.3983

**Published:** 2021-06-18

**Authors:** Mahmoud S. Eisa, Mustafa A. Al‐Tikrity, Mona M. Babikikir, Mohamed A. Yassin

**Affiliations:** ^1^ Hamad Medical Corporation Doha Qatar; ^2^ Internal Medicine Residency Program Hamad General Hospital Hamad Medical Corporation Doha Qatar; ^3^ Hematology Department National Center for Cancer Care and Research Hamad Medical Corporation Doha Qatar

**Keywords:** anemia, iron, iron deficiency anemia, thrombocytopenia

## Abstract

A broad spectrum of diseases can cause anemia and thrombocytopenia. Some of these diseases are a hematological emergency; others are benign diseases, so early and accurate diagnosis is crucial in managing such patients. Usually, IDA is associated with thrombocytosis or normal platelets; however, in rare cases, IDA can be associated with thrombocytopenia; even though, thrombocytopenia that occurs with IDA responds to iron therapy. Iron therapy rarely causes transient thrombocytopenia per se. We are reporting an African female patient who is found to have thrombocytopenia secondary to iron deficiency anemia (IDA), and she responded to iron replacement therapy initially with a transient drop in platelets, followed by a rapid rise in platelets till platelets reached the normal level.

## Graphical abstract

Iron deficiency anemia can be associated with thrombocytopenia. It should be thought of after ruling out other differential diagnosis. It responds to iron replacement therapy, which can cause a transient drop in platelets initially then normalize.

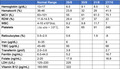

## 1 INTRODUCTION

Anemia is a global public health problem that affects up to 49% of the population worldwide. About 29% of all women of reproductive age have anemia globally.^1^ Approximately 50% of cases of anemia are considered to be due to iron deficiency, but the proportion probably varies among population groups and in different areas.^2^

The anemia cutoffs vary based on age, sex, and pregnancy‐specific (Table 1). Severe anemia is defined by WHO as Hb < 70 g/L in pregnant women and children under 5 years of age and Hb < 80 g/L in all other age groups.^3^

**TABLE 1:** Hemoglobin (g/L) concentrations to diagnose anemia at sea level^3^

Population	Nonanemic	Anemia		
		Mild	Moderate	Severe
Children 6‐59 mo of age	≥110	100‐109	70‐99	<70
Children 5‐11 y of age	≥115	110‐114	80‐109	<80
Children 12‐14 y of age	≥120	110‐119	80‐109	<80
Nonpregnant women (15 y of age and above)	≥120	110‐119	80‐109	<80
Pregnant women	≥110	100‐109	70‐99	<70
Men (15 y of age and above)	≥130	110‐129	80‐109	<80

In chronic anemia usually, the body accumulates, and patients might be asymptomatic or have mild symptoms; on the other hand, acute anemia typically presents with more apparent symptoms.^4^

The quality of life can be affected by IDA as previously reported to affect the metabolism of glucose, thyroid function, and spermatogenesis.^5^, ^6^, ^7^

Iron deficiency anemia is reported to affect other blood parameters, for example, neutropenia and lymphocytopenia.^8^

The association between IDA and platelet is complex; iron deficiency is usually associated with either normal platelet counts or thrombocytosis. In rare conditions, IDA can be associated with thrombocytopenia, and there if IDA corrected the thrombocytopenia correct concurrently.^9^ Rarely, with the correction of IDA, some patients develop transient neutropenia.^10^

Anemia and thrombocytopenia can be seen together in various diseases; some of these diseases need urgent intervention, such as thrombotic microangiopathy and bone marrow replacement disorders, for example, leukemia; others are cold cases such as paroxysmal nocturnal hemoglobinuria, Evan's syndrome, and aplastic anemia, so it is always challenging to narrow the differential diagnosis early upon patient presentation as early intervention in some of this diseases has mortality benefit like in the case of thrombotic microangiopathy and leukemia. Blood peripheral smear is always the first step that usually can guide the management plan.

Heavy menstrual bleeding (HMB) is a common gynecologic problem that affects around 27% of women. Chronic heavy or prolonged uterine bleeding is a common cause of severe anemia in women.^11^

## 2 CASE REPORT

A 32‐Year‐old Kenyan female patient not known to have any chronic illness admitted to our institute in August 2019 with the chief complaints of colicky abdominal pain for 2 days; she reported tiredness, fatigue, and shortness of breath that worsen with exertion. There was no associated nausea, vomiting, change in bowel habit, weight loss, or fever.

She gave a history of heavy menstrual bleeding for the past 2 years, and she has no family history of chronic diseases or anemia. Social history was unremarkable for smoking, alcohol drinking, and she was not sexually active. She works as a maid.

Clinical examination was remarkable for pallor only, with unremarkable abdominal as well as other general examination.

Initial laboratory workup for her revealed anemia with hemoglobin 6.5 g/dL(13‐17 g/dL), platelet count 54 000 × 10^9^/L (150 000‐450 000 × 10^9^/L). Peripheral blood smear revealed a dimorphic blood picture with the majority of cells markedly hypochromic and microcytic. Iron profile confirmed the IDA picture with low iron, ferritin levels, and elevated TIBC level.

After confirming the results, the patient received intravenous 750 mg of ferrous carboxy maltose based on her iron profile, and in the second day, after ruling out TTP by blood peripheral smear findings, one unit of packed red blood cells transfused to the patient after that patient symptoms improved. Her platelet count followed on days 3 and 4 which showed improvement to 65, 91 × 10^9^/L, respectively. Another platelet count followed after 50 days from discharge which was 240 × 10^9^/L. No further workup was done as the patient symptoms and condition improved rapidly.

## 3 DISCUSSION

We are describing a young adult African woman who was found to have severe iron deficiency anemia and thrombocytopenia. Iron deficiency anemia in this patient was mostly attributed to her heavy menstrual bleeding as well as possible nutritional deficiency which can be from her economic status as she works as a maid. The patient presentation and symptoms was related to the anemia, and her anemia symptoms were moderate despite having low hemoglobin "grade 4 anemia,^12^ all of this is pointing toward that this anemia is chronic.^9^

Iron deficiency anemia is usually associated with either normal platelets or thrombocytosis. The association between IDA and thrombocytopenia is rare. And it is best diagnosed retrograde after correcting the anemia; the platelets will rise.

The exact mechanism of IDA and thrombocytopenia is not well understood; Apar Kishor Ganti et al suggested that it may be related to the alteration in the activity of iron‐dependent enzymes in thrombo‐ and leukopoiesis.^13^ Another suggested mechanism by T P McDonald and R E Clift Cottrell might be an early response to direct stimulation of the EPO receptor on megakaryocytes or shunting into the erythroid precursor pathway, leading to decreased platelet formation.^14^

The peripheral smear of the patient revealed a dimorphic blood picture with the majority of cells markedly hypochromic and microcytic was another reassuring finding toward IDA as a likely related cause for her thrombocytopenia, which in other patients with fragmented RBCs or abnormal WBCs morphology needs further investigations to rule out serious problems.

Based on the history, clinical examination, Hb, iron study, and peripheral smear picture, the patient was started on treatment for iron deficiency anemia by IV iron and transfusion of one unit of packed RBCs. After the patient received the IV iron, her platelets counts dropped transiently for 2 days and then started to pick up rapidly after 3‐4 days. A follow‐up clinic with laboratory result after 50 days from discharge showed platelet count within normal range 240 × 10^9^/L, and the patient was completely Asymptomatic Table 2.

**TABLE 2:** Complete blood count and iron profile before and after iron transfusion

	Normal range	at admission	4 days after admission	5 days after admission	after 2 months
Hemoglobin (g/dL)	13‐17	6.5	9.1	8.5	12
Hematocrit (%)	36‐46	23.8	32	29	41.5
MCV (fL)	83‐101	55	61	61.3	70.1
RDW (%)	11.6%‐14.5%	29.4	37	37	22
WBC	4‐10 × 10^3^/uL	9.2	9.8	11.7	7
Platelets	150‐450 × 10^9^/L	54	65	91	240
Reticulocytes (%)	0.5‐2.5	0.6		1.9	0.8
Iron (μg/dL)	6‐35	6			9
TIBC (μg/dL)	45‐80	95			68
Transferrin (gm/L)	2.0‐3.6	3.8			2.7
Ferritin (ng/mL)	6.0‐44.0	5.2			16.7
Folate (ng/mL)	2‐20	17.8			16.9
LDH (U/L)	125‐220	225			
Vitamin B12 (pg/mL)	160‐950	322			

## 4 CONCLUSION

Iron deficiency anemia can be associated with thrombocytopenia. It should be thought of after ruling out serious differential diagnosis like TTP; thrombocytopenia caused by IDA responds to iron replacement therapy, which can cause a transient drop in platelets initially.

## CONFLICT OF INTEREST

None declared.

## AUTHOR CONTRIBUTIONS

MSE and MAA‐T: took the lead in writing the manuscript, literature review, and created the legends. MAA‐T and MAY: revised manuscript critically for important intellectual content. All took care of the patient, contributed to and approved the final version of the manuscript.

## ETHICAL APPROVAL

Ethical approval for this study was obtained from The Medical Research Center At Hamad Medical Corporation (ABHATH) ID: MRC‐04‐20‐445.

## Data Availability

Individual data for this patient will not be publicly available due to ethical restrictions by HMC medical research center that does not generally allow sharing of data to individuals or entities outside Hamad Medical Corporation.
